# Transcriptome-wide Analysis of Exosome Targets

**DOI:** 10.1016/j.molcel.2012.08.013

**Published:** 2012-11-09

**Authors:** Claudia Schneider, Grzegorz Kudla, Wiebke Wlotzka, Alex Tuck, David Tollervey

**Affiliations:** 1 Wellcome Trust Centre for Cell Biology, The University of Edinburgh, Edinburgh UK

## Abstract

The exosome plays major roles in RNA processing and surveillance but the in vivo target range and substrate acquisition mechanisms remain unclear. Here we apply in vivo RNA crosslinking (CRAC) to the nucleases (Rrp44, Rrp6), two structural subunits (Rrp41, Csl4) and a cofactor (Trf4) of the yeast exosome. Analysis of wild-type Rrp44 and catalytic mutants showed that both the CUT and SUT classes of non-coding RNA, snoRNAs and, most prominently, pre-tRNAs and other Pol III transcripts are targeted for oligoadenylation and exosome degradation. Unspliced pre-mRNAs were also identified as targets for Rrp44 and Rrp6. CRAC performed using cleavable proteins (split-CRAC) revealed that Rrp44 endonuclease and exonuclease activities cooperate on most substrates. Mapping oligoadenylated reads suggests that the endonuclease activity may release stalled exosome substrates. Rrp6 was preferentially associated with structured targets, which frequently did not associate with the core exosome indicating that substrates follow multiple pathways to the nucleases.

## Graphical abstract

**Graphical Abstract:**

**Figure:**
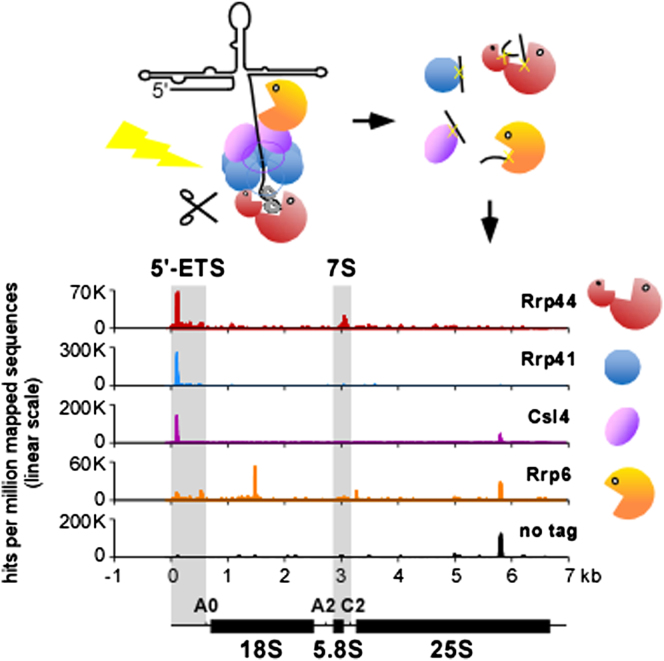

**Highlights:**

► The in vivo target range was identified for the exosome nuclease complex ► The endonuclease and exonuclease activities of Rrp44/Dis3 function cooperatively ► Comparison of Rrp6 and core exosome suggests multiple substrate recruitment pathways ► Pre-tRNA and other RNAs transcribed by Pol III emerge as major exosome targets

## Introduction

Gene expression generates an enormous variety of stable or unstable, protein-coding or non-coding RNA species produced by all three RNA polymerases. RNA abundance and integrity are closely monitored by nuclear and cytoplasmic surveillance systems (reviewed in (Houseley and Tollervey, 2009)). A key player in RNA metabolism is the exosome, which participates in 3′ end maturation and/or quality control of almost every RNA molecule in the cell. In *Saccharomyces cerevisiae*, nuclear and cytoplasmic forms of the exosome share the RNase II homolog Rrp44/Dis3, which contains two distinct catalytic sites. The RNB domain exhibits 3′-5′ exonuclease activity, whereas the N-terminal PINc domain plays a dual role in harboring endonuclease activity and tethering Rrp44 to the core, nine subunit exosome (Lebreton et al., 2008; Lorentzen et al., 2008; Schaeffer et al., 2009; Schneider et al., 2009). In addition to Rrp44, the nuclear form of the yeast exosome is associated with a second active 3′-5′ exonuclease, Rrp6 (Briggs et al., 1998).

Structural studies have shown that the nine catalytically inert subunits of the core exosome form a two-layered barrel-like structure (Liu et al., 2006). The upper layer is composed of a “cap” of three S1 or KH domain RNA binding proteins (Csl4, Rrp4, Rrp40), which rests on a ring of six proteins with homology to RNase PH (Rrp41, Rrp45, Rrp43, Rrp46, Rrp42 and Mtr3). Rrp44 is located at the base of the core exosome barrel, and in vitro data show that substrates can be threaded through the lumen of the exosome barrel to reach the exonuclease site in Rrp44 (Bonneau et al., 2009; Malet et al., 2010). However, it is not known what fraction of natural substrates follow this path. Rrp6 has distinct targets (Callahan and Butler, 2008) and associates with the exterior of the exosome complex.

Vital functions of the exosome include the processing of ribosomal RNA (rRNA), small nuclear and nucleolar RNAs (sn(o)RNAs) in the nucleus, mRNA turnover in the cytoplasm and surveillance of aberrant RNAs throughout the cell (reviewed in (Houseley and Tollervey, 2009)). It also plays key roles in the regulated degradation of pervasive transcripts that are generated all over the yeast genome. These include cryptic unstable transcripts (CUTs), which were originally identified in strains lacking Rrp6, stable un-annotated transcripts (SUTs) and many short, promoter-associated RNAs (PARs) (Davis and Ares, 2006; Neil et al., 2009; Wyers et al., 2005; Xu et al., 2009). Distinct classes of RNA substrates are likely assigned to individual nuclease activities in the exosome, but substrate specificities and targeting mechanisms for this process are largely unclear. Microarray analyses have been applied to distinguish substrate specificities of Rrp6, Rrp44/Dis3, and core exosome subunits in *Drosophila*, but this was limited to mRNAs (Kiss and Andrulis, 2010).

Most functions of the exosome are dependent on cofactors, including the Trf-Air-Mtr4 polyadenylation (TRAMP) complex and the Nrd1/Nab3 heterodimer, but direct interactions between individual exosome subunits and some specific targets have been reported (Kadaba et al., 2006; Schneider et al., 2007). Transcriptome-wide maps of RNA substrates of the TRAMP-associated poly(A) polymerase Trf4 or the Nrd1/Nab3 heterodimer have been published based on UV crosslinking (Jamonnak et al., 2011; Wlotzka et al., 2011) or RNA coprecipitation (Hogan et al., 2008; San Paolo et al., 2009). These analyses identified many surveillance targets, including a notable number of RNA polymerase III transcripts.

Here we report a transcriptome-wide map of exosome substrates and their interactions with individual exosome subunits in living cells.

## Results

### Comparison of Targets for Wild-Type and Mutant Forms of Rrp44

**Figure 1:**
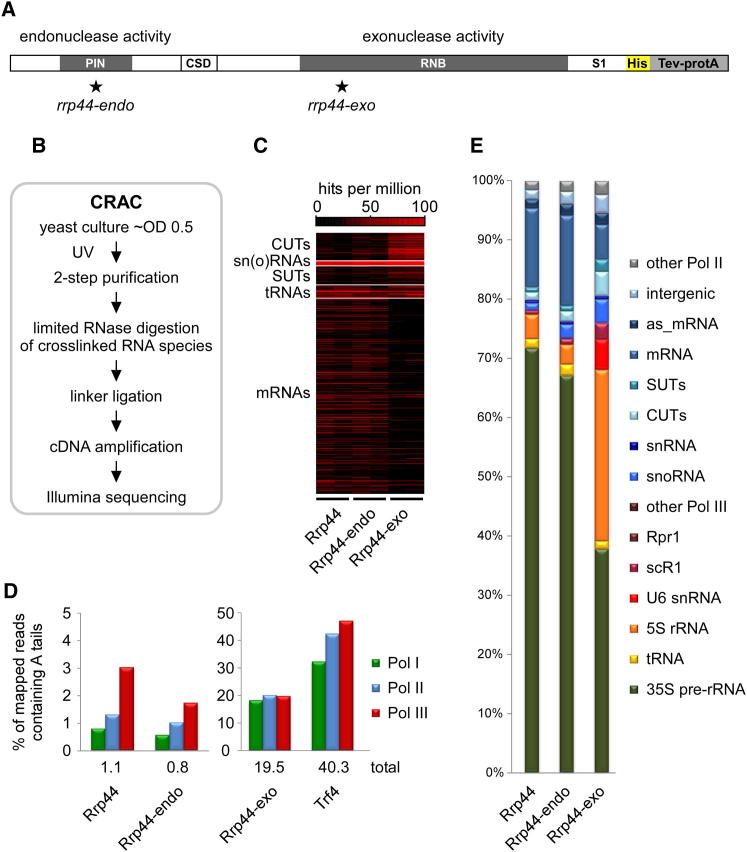
Comparison of Targets of Wild-Type and Mutant Rrp44 (A) Domain structure of *S. cerevisiae* Rrp44, including a C-terminal His-TEV protease-protein A (HTP) tag for purification. Point mutations inactivating the endonuclease (*rrp44-endo*) or exonuclease (*rrp44-exo*) activity of Rrp44 are indicated. (B) Outline of the CRAC crosslinking technique. (C–E) Illumina high-throughput sequencing of cDNA libraries generated from crosslinked RNAs recovered with purified wild-type Rrp44 and the Rrp44-endo and Rrp44-exo mutants, as well as the exosome cofactor Trf4. Here, and in all other illustrations, sequencing data of individual biological replicate experiments was mapped to the yeast genome using Novoalign and normalized to hits per million mapped sequences (hpm). (C) Heat maps for main substrate groups. Numbers of reads mapped to individual RNAs are shown in shades of red. (D) Frequencies of non-templated terminal oligo(A) sequence reads in data sets for wild-type Rrp44 and catalytic mutants, and the exosome cofactor Trf4. Data sets are filtered either for total reads, or for Pol I, Pol II and Pol III transcripts, that contain 2 or more non-templated As. (E) Transcriptome-wide binding profiles. Bar diagrams illustrate the percentage of all sequences mapped to the functional RNA classes indicated on the right of the figure.

To identify targets for the core exosome nuclease Rrp44, we applied in vivo RNA-protein crosslinking (CRAC) (Granneman et al., 2009) to the wild-type protein, or *rrp44* mutants carrying point mutations in catalytic residues of the RNB exonuclease domain (*rrp44-exo* mutant, D_551_N) or PIN endonuclease domain (*rrp44-endo* mutant, D_91_N, E_120_Q, D_171_N, D_198_N) (Figure 1A). HTP-tagged forms of Rrp44 were expressed from a plasmid in yeast strains derived from BY4741, in which the genomic *RRP44* ORF was precisely deleted. Growth rates and RNA processing phenotypes of strains expressing either wild-type or mutant Rrp44 were as previously reported (Schneider et al., 2009). Cells actively growing in minimal SD medium were UV-irradiated as described (Granneman et al., 2011) and RNA fragments crosslinked to Rrp44 were identified by the CRAC technique as outlined in Figure 1B. At least two independent experiments were performed in each case and analyzed separately. The primary sequence data have been deposited in NCBI’s Gene Expression Omnibus (Edgar et al., 2002) and are accessible through GEO Series accession number GSE40046. Mapped reads are presented in Table S3.

Transcriptome-wide binding profiles of Rrp44 are shown in Figures 1C–1E. Wild-type and mutant forms of Rrp44 were predominately associated with classes of RNA corresponding to known exosome targets. Analysis of individual, functionally grouped RNAs (Figures 1C, S1, and Table S3) revealed similar patterns for wild-type Rrp44 and Rrp44-endo data sets. The *rrp44-endo* mutation does therefore not appear to significantly alter or interfere with Rrp44 substrate binding. In contrast, the Rrp44-exo data set was significantly enriched for sequences derived from CUTs, SUTs, snRNAs, snoRNAs and, most prominently, a subset of Pol III RNAs (5S rRNA, U6 snRNA, scR1), whereas recovery of mRNAs and the 35S pre-rRNA was relatively reduced.

The initial identification of CUTs in strains lacking only Rrp6 (Davis and Ares, 2006; Wyers et al., 2005) had suggested that Rrp6 was the major nuclease responsible for their degradation. However, the enrichment for CUTs in Rrp44-exo data sets strongly indicates that CUTs are also targeted for degradation by Rrp44.

The presence of non-templated, 3′ terminal oligo(A) tails is a characteristic of nuclear RNA surveillance targets (reviewed by (Houseley and Tollervey, 2009)). The Trf4-HTP data set generated here from actively growing cells contained a high fraction (40.3%) of reads with ≥ 2 non-templated adenosines at the 3′ end (Figure 1D). In contrast, few oligoadenylated reads were recovered in wild-type Rrp44 (1.1%) or Rrp44-endo (0.8%) data sets, and such reads were predominately derived from Pol III transcripts (Figure 1D). However, for the Rrp44-exo mutant 19.5% of mapped sequences derived from all three polymerases carried an oligo(A) tail, indicating that Rrp44-exo becomes trapped on degradation intermediates of the targets of nuclear RNA surveillance. To characterize RNA targets associated with wild-type and mutant forms of Rrp44, we initially compared the distribution of mapped sequences among different substrate classes (Figure 1E). All three data sets contain a large percentage of sequences mapped to the Pol I transcribed 35S pre-rRNA, reflecting the prominent roles of Rrp44 and the exosome in ribosome biogenesis and pre-rRNA surveillance. Both stable and unstable non-coding RNAs transcribed by RNA polymerases II and III, as well as a large pool of (pre-)mRNAs, were also crosslinked to all Rrp44 variants.

**Figure 2:**
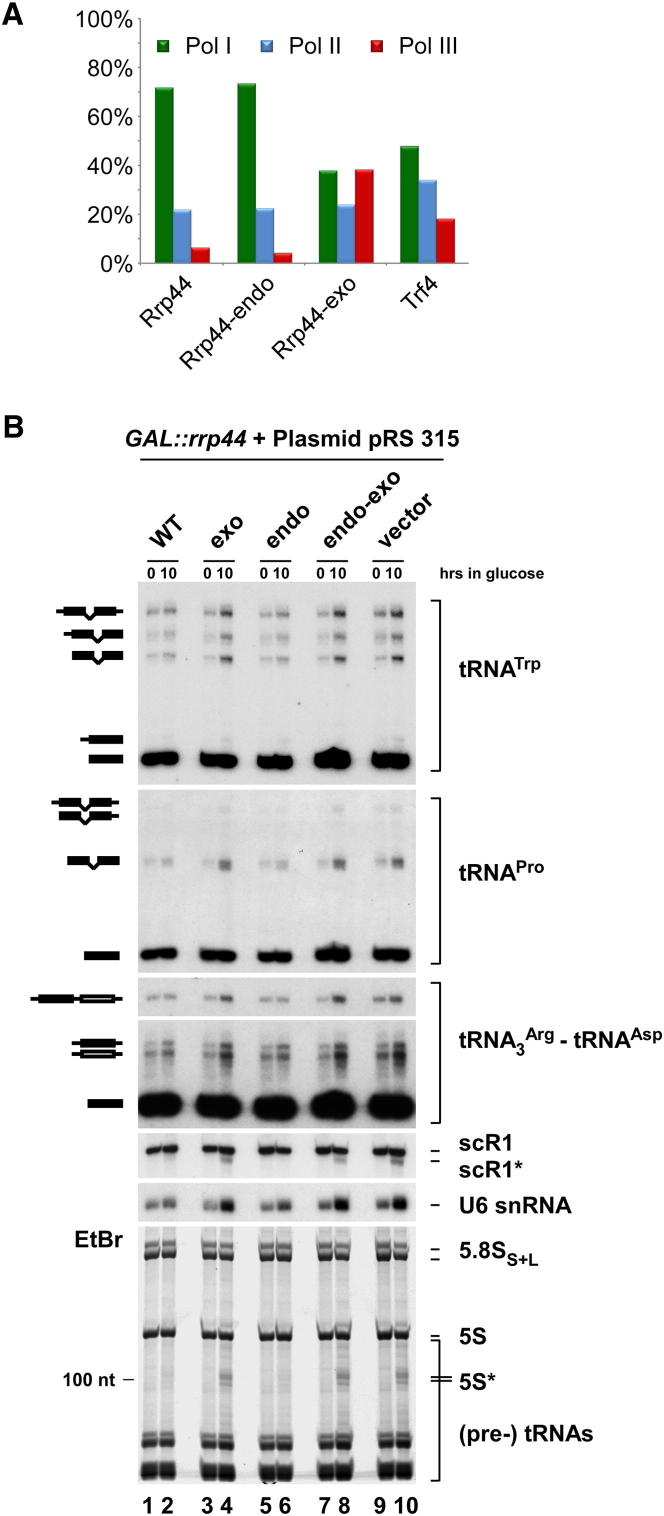
The Exosome and Cofactors Target RNA Pol III Transcripts (A) Proportion of reads mapped to products of RNA Polymerases I, II and III recovered with wild-type Rrp44 and catalytic mutants, and with the exosome cofactor Trf4. (B) Northern analyses showing pre-tRNA and other Pol III RNA accumulation in strains expressing Rrp44 mutants. A *GAL::rrp44* strain was transformed with plasmids expressing either wild-type or mutant Rrp44 protein, or an empty vector pRS315. The mutants analyzed are Rrp44-exo, Rrp44-endo and Rrp44-endo-exo (see Figure 1A). RNA was isolated from strains grown at 30°C under permissive conditions (0) and 10 hr after transcriptional repression (10). RNA was separated on an 8% polyacrylamide/ 8M urea gel and either detected by northern hybridization with oligonucleotide probes (Table S1) or by staining with EtBr. A schematic representation of the identified species is shown.

A striking feature of the Rrp44-exo data set was the abundant recovery of Pol III RNAs (Figures 2A and S2A). While such transcripts represent only ∼5% of all RNAs recovered with wild-type Rrp44 or Rrp44-endo, almost 40% of all RNAs crosslinked to Rrp44-exo are transcribed by Pol III. RNAs transcribed by Pol III also comprised a substantial proportion (18.2%) of the Trf4 data set (Figures 2A and S2A), consistent with crosslinking (San Paolo et al., 2009; Wlotzka et al., 2011) and experimental data implicating Trf4 in the surveillance of 5S rRNA, U6 snRNA and pre-tRNAs (Copela et al., 2008; Kadaba et al., 2004; Kadaba et al., 2006; Schneider et al., 2007; Schneider et al., 2009; Vanácová et al., 2005; Wlotzka et al., 2011).

The prominent association of Pol III targets with Rrp44-exo and nuclear exosome cofactors indicates that these are major targets for the nuclear RNA surveillance machinery. The reduced recovery of Pol III transcripts with Rrp44 and Rrp44-endo suggests that they are substrates for the Rrp44 exonuclease activity, but are normally degraded efficiently. The high proportion of oligoadenylated sequences derived from Pol III RNAs in the Rrp44-exo data sets shows that the crosslinked RNAs had already been targeted and marked for surveillance (shown for the U6 snRNA, 5S rRNA and tRNA^Pro^ in Figures S2B–S2D). Persistent binding of these RNAs to the exosome in the absence of Rrp44 exonuclease activity may contribute to the impaired growth and strong RNA processing phenotypes in *rrp44-exo* strains.

To further assess the role of the distinct catalytic activities of Rrp44 in Pol III RNA surveillance in vivo, levels of pre-tRNAs and other Pol III transcripts were assessed in Rrp44 mutant strains (Figure 2B). For this, the endogenous Rrp44 was expressed as HA-fusion under the control of a repressible *P*_*GAL10*_ promoter. The strain was then transformed with plasmids expressing Rrp44, Rrp44-endo, Rrp44-exo or Rrp44-endo-exo with a C-terminal Protein A tag and under the control of the *P*_*RRP44*_ promoter (Schneider et al., 2009), or with the empty cloning vector (pRS315). Changes in Pol III RNA levels were observed in strains expressing Rrp44-exo, whereas the Rrp44-endo mutation alone had no effect. Rrp44-exo phenotypes included pre-tRNA accumulation and the appearance of 3′ truncated fragments (5S^∗^, scR1^∗^). Higher levels of mature U6 snRNA were seen and 3′ extended (∼3nt) U6 was observed in the sequence data (data not shown), consistent with a gel mobility shift (Figure 2B). We conclude that the exonuclease activity of Rrp44 plays multiple roles in the surveillance and/or maturation of Pol III transcribed RNAs.

### Split-CRAC Separates Targets for the Exonuclease and Endonuclease Domains of Rrp44

**Figure 3:**
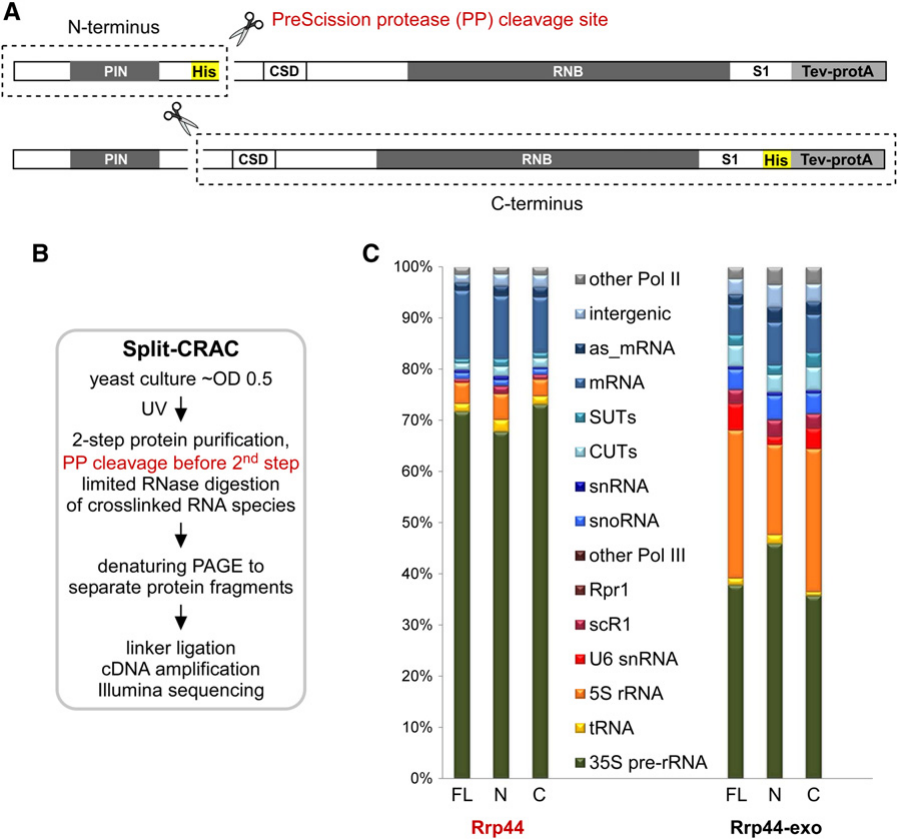
Split-CRAC Allows the Targets of the N-Terminal and C-Terminal Regions of Rrp44 To Be Distinguished (A) Cleavable Rrp44-expression constructs used for split-CRAC. The location of the PreScission protease (PP) cleavage site, which allows the separation of N- and C-terminal domains (NTD and CTD) in vitro, and the purification tags are indicated. (B) Outline of the split-CRAC crosslinking technique. (C) Distribution of reads recovered with full-length and cleaved wild-type Rrp44 (left) and the Rrp44-exo mutant (right). Sequencing data were analyzed as in Figure 1.

Many proteins that function in RNA metabolism contain more than one RNA interacting domain, but determining their relative contributions in vivo can be experimentally challenging. In the case of Rrp44 we wanted to assess the possibility that the PIN and RNB domains might specifically and independently contribute to RNA target recognition in vivo. To identify RNAs preferentially associated with each of the two domains, we developed a modified CRAC protocol, termed split-CRAC (Figures 3A and 3B). In this, the intact protein is crosslinked in vivo in actively growing cells, followed by in vitro cleavage during protein purification.

Rrp44 expression plasmids were constructed in which a PreScission protease (PP) cleavage site was inserted between aa 241 and aa 242 of the *RRP44* or *rrp44-exo* ORF. This insertion site was chosen because structural studies on Rrp44 had previously shown that a C-terminal fragment truncated at aa 242 was stable (Lorentzen et al., 2008), indicating that the protein domain structure was unlikely to be perturbed by the short insert. The constructs also carry a His_6_ tag, located on either the N-terminal or C-terminal side of the cleavage site (Figure 3A). Cleavage of crosslinked Rrp44-RNA complexes during purification was shown to allow selective recovery of either the N-terminal domain (NTD) or C-terminal domain (CTD) dependent on the location of the His_6_ tag (Figure S3).

**Figure 4:**
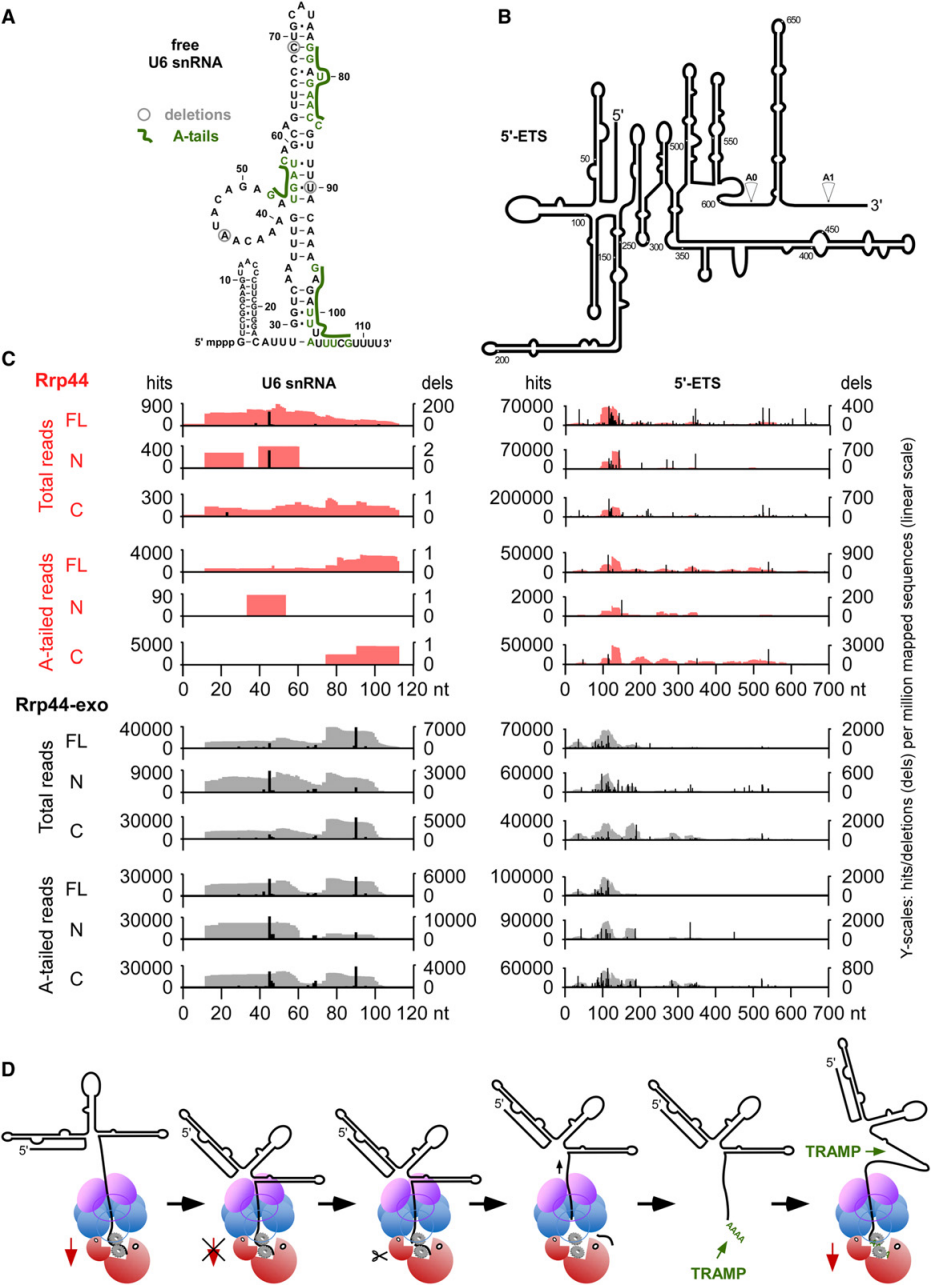
Comparison of Rrp44 Domains in Split-CRAC (A) Secondary structure of U6 snRNA (112 nt) from *S. cerevisiae*. Major sites of microdeletions are circled. These are due to reverse transcriptase stops at the crosslinked nucleotide. Prominent sites of oligoadenylation are indicated in green and are located 3′ to the major crosslinking sites. The positions of the first non-templated adenosines in A-tailed reads are indicated in green. (B) Predicted secondary structure for the pre-rRNA 5′-ETS region (699 nt) from *S. cerevisiae*. Processing sites A0 and A1 are indicated by arrowheads. (C) Read coverage for full-length and cleaved Rrp44 (red) and Rrp44-exo (black) in the U6 snRNA (left) and the pre-rRNA 5′-ETS region (right). Mapped reads (hits) are depicted in red (Rrp44) or gray (Rrp44-exo); positions of microdeletions (dels) are indicated in black. Data sets used for analysis were either unfiltered (Total) or filtered for reads containing 2 or more non-templated As (A-tailed). FL – full-length protein; N – NTD; C – CTD. (D) Proposed model for the cooperative action of the endonuclease and exonuclease activities of Rrp44 and the TRAMP complex on structured RNA substrates. Many substrates are threaded through the exosome barrel to reach the active sites of Rrp44, which interacts with the exosome core via the NTD (Bonneau et al., 2009; Malet et al., 2010; Schaeffer et al., 2009; Schneider et al., 2009). Proteins of the RNase II family, which includes the exonuclease domain of Rrp44, are strongly processive and bind substrates tightly in the active site cleft (Zuo et al., 2006). This presumably allows Rrp44 to actively pull substrate RNAs in through the complex (1). However, only single stranded RNAs can enter the lumen of the exosome, so stable RNA-RNA or RNA-protein structures in the substrate potentially lead to stalled complexes, in which the 3′ end is tightly but non-productively bound by Rrp44 (2). We postulate that under these circumstances, the PIN domain cleaves the RNA (3), allowing substrate release (4). The substrate could then be re-adenylated by the TRAMP complex (5) and reloaded into the exosome (6), probably with the assistance of TRAMP.

The transcriptome-wide interaction profiles were strikingly similar for the full-length (FL) Rrp44 protein and for both the NTD and CTD fragments (Figure 3C). Using the Rrp44-exo mutant in split-CRAC, the analyses also returned very similar overall patterns of hits for the full-length protein compared to either fragment. The only exceptions were decreased recovery of the U6 snRNA and the 5S rRNA with the NTD and mildly decreased pre-tRNAs with the CTD. However, more detailed analyses of the hit distribution across individual target RNAs with high sequence coverage revealed differences in binding profiles. This is illustrated for the U6 snRNA (Figure 4A) and the pre-rRNA 5′-External Transcribed Spacer (5′-ETS) region (Figure 4B).

In CRAC and related techniques, microdeletions are often introduced at the site of crosslinking during reverse transcription and can be used to precisely map protein binding sites (Wlotzka et al., 2011; Zhang and Darnell, 2011). To distinguish the relative positions of the NTD and CTD on target RNAs at higher resolution, we compared the mapped reads (hits) of Rrp44 and Rrp44-exo split-CRAC data sets with the positions of microdeletions (dels) (Figure 4A–4C). These analyses were performed using the complete data set (Total reads) and also following filtering for sequences that contain oligo(A) tails (A-tailed reads), to identify RNAs that the TRAMP complexes have marked for degradation.

In the U6 snRNA, different binding profiles are seen for the NTD PINc and CTD exonuclease regions of Rrp44 (Figure 4C, left panel). The NTD mainly binds at the 5′ end of the RNA, whereas CTD hits are shifted toward the 3′ end. The same pattern is seen for total and A-tailed reads, indicating that the recovered fragments are largely derived from RNAs that were targeted for surveillance. The distribution of reads and deletions in the Rrp44-exo mutant generally matches this pattern but the higher recovery of U6 snRNA sequences with microdeletions allows better mapping of the crosslinking sites for the NTD around nt 45, for the CTD around nt 90, and for all constructs around nt 69. Notably, the number of deletions in the A-tailed reads of the NTD increased relative to the CTD (Figure 4C). These transcripts must have been released from the exosome complex, and then reinserted, in order for oligo(A) addition by TRAMP to have occurred.

The highly structured 5′-A_0_ fragment of the 5′-ETS is a well-characterized exosome substrate, which is very rapidly and “constitutively” degraded as part of the pre-rRNA processing pathway (Lebreton et al., 2008; Schaeffer et al., 2009; Schneider et al., 2009). In the 5′-ETS, both wild-type and mutant full-length Rrp44 and fragments bound in the region around nt 120, but differences in the distribution of hits and deletions were seen for the fragments along the whole RNA (Figure 4C, right panel). For Rrp44-exo, the differences in the distribution of reads were more pronounced and the coverage of NTD reads was increased relative to the (catalytically inactive) CTD. Coverage is expressed in hits/dels per million mapped reads, so this reflects a change in the relative distributions of the Rrp44-exo domains over all substrates. While absolute crosslinking efficiencies cannot be reliably inferred, the yield of crosslinked RNAs was reproducibly higher in the Rrp44-exo strain than in Rrp44 (see Figure S3), consistent with prolonged interactions leading to increased crosslinking efficiency.

The major peaks across the 5′-ETS represent structured regions, where exosome pausing may occur (Lebreton et al., 2008). We postulate that endonuclease cleavage acts to release stalled RNAs that are tightly bound by the exonuclease site of Rrp44 (Figure 4D). Oligoadenylation of the released substrate by Trf4 may allow the TRAMP-associated helicase Mtr4 to unwind the structured RNA, which can then be threaded back through the exosome channel.

More generally, split-CRAC can distinguish domain-specific interactions and should be widely applicable to resolve the targets of multi-domain RNA-binding proteins – which are common.

### Comparison of RNA Targets for Core Exosome Subunits and Rrp6

**Figure 5:**
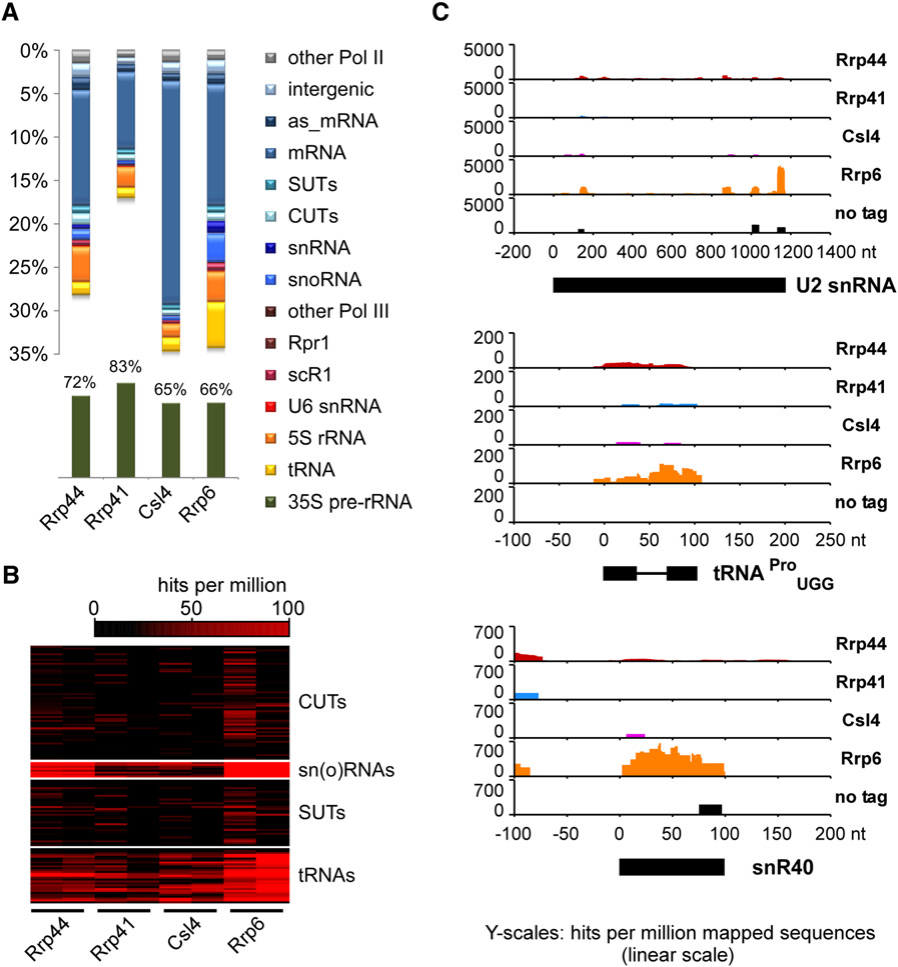
Comparison of Targets of the Core Exosome and Rrp6 (A) Proportion of sequences corresponding to functional RNA classes recovered with core exosome subunits (Rrp44, Rrp41, Csl4), and Rrp6. Sequencing data was analyzed as in Figure 1. (B) Heat maps for main non-protein coding RNA substrate groups recovered with the indicated IP. Numbers of reads mapped to individual RNAs are shown in shades of red. (C) Read coverage along highly structured RNAs (top) the U2 snRNA (*LSR1*; 1175nt); (middle) pre-tRNA^Pro^_UGG_ (102nt, intron 37-66nt) and (bottom) the box C/D snoRNA snR40 (97nt).

Yeast Rrp44 is present in the exosome throughout the cell, whereas Rrp6 is only associated with the nuclear complex. To assess the relationship between Rrp6 and the core exosome, we constructed yeast strains expressing C-terminal tagged Rrp6-HTP, Rrp41-HTP or Csl4-HTP, each expressed from the endogenous genomic locus under the control of the endogenous promoter. Rrp41 forms part of the exosome barrel, which is composed of six RNase PH-like proteins, whereas the S1 RNA-binding domain protein Csl4 is one of the three “cap” proteins at the top of the barrel (Liu et al., 2006). All strains showed wild-type growth rates, indicating that the fusion proteins were functional. CRAC was performed as for Figure 1 and crosslinking patterns of core exosome subunits (Rrp44, Rrp41, Csl4) are compared to Rrp6 in Figure 5. Core exosome targets showed a high degree of overlap, with similar distributions of hits on most RNA classes, although some variation in recovery of pre-mRNA and mRNA was observed (Figures 5A, 5B, and S1). In contrast, the Rrp6 data sets were relatively enriched in small, structured RNAs including tRNAs, snRNAs and snoRNAs (Figures 5A and 5B). These were analyzed in more detail and representative results are presented for three examples; the U2 snRNA, the intron-containing pre-tRNA^Pro^_UGG_ and the box C/D snoRNA snR40 (Figure 5C). All three targets show higher coverage for Rrp6 than for Rrp44, and very low for Csl4 or Rrp41. In the case of snR40, the reads detected for Rrp6 are distributed over the body of the RNA, although some 3′ extended reads were observed in one experiment. We interpret this as indicating a major role for Rrp6 in surveillance and degradation, rather than in 3′ end processing of pre-snR40.

The crosslinking data also provided examples of functional overlap between Rrp6 and Rrp44, but not the remaining core exosome components, on structured RNAs. This is shown for the box C/D snoRNA snR13 (Figure S4). Rrp6 and Rrp44 are both required for the 3′ end processing of this RNA, while *Δrrp6* strains also accumulate snR13 read-through transcripts (Grzechnik and Kufel, 2008; Schneider et al., 2009). These aberrant RNAs are hardly visible in *rrp44-exo* strains, but strongly enriched in *Δrrp6 rrp44-exo* double mutants compared to the *Δrrp6* strain (Schneider et al., 2009). Interestingly, Rrp6 was mainly crosslinked to the highly structured mature snR13 RNA region, whereas sequences recovered with Rrp44 and, in particular, Rrp44-exo were often derived from downstream regions (Figure S4). As seen for other structured RNAs (Figure 5), few sequences were recovered for Rrp41 or Csl4 on snR13 (Supp. Data set). The in vivo analyses and crosslinking data both suggest that transcriptional read-though mainly occurs following defective 3′ end formation on snR13, with the resulting 3′ extended transcripts being targeted to Rrp44 for degradation.

**Figure 6:**
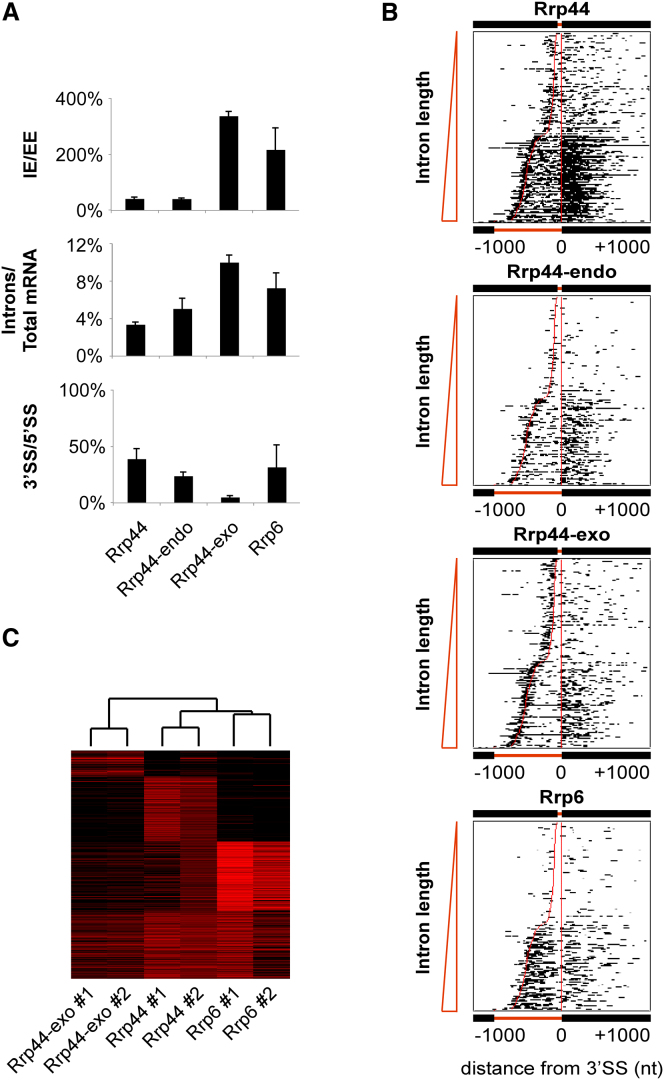
Interactions of Rrp44 and Rrp6 with Pre-mRNA (A) Frequencies of reads mapped to pre-mRNAs and mature mRNAs. IE/EE: Relative numbers of reads mapped to intron-exon junctions (IE) in pre-mRNAs relative to exon-exon junctions (EE) in mature mRNAs. Introns/Total mRNA: Numbers of reads mapped to mRNA introns relative to the total number of reads mapped to mRNAs. 3′SS/5′SS: Relative numbers of reads mapped to 3′ splice sites (3′SS) in pre-mRNAs, relative to 5′ splice (5′SS) junctions. Bars indicate the standard error. (B) Rrp44 and Rrp6 binding profiles (black) along 219 intron-containing pre-mRNAs. Pre-mRNAs are aligned at their 3′ splice sites, and ordered by intron length. Intron boundaries are shown as red lines. (C) Grouping of 4849 mRNAs by pattern of interactions with exosome proteins. Experiments were clustered by complete linkage using the correlation distance metric. Replicate experiments clustered together confirming the reproducibility of the data. Numbers of reads mapped to individual RNAs are shown in shades of red.

The core exosome including Rrp44 plays major roles in surveillance and turnover of cytoplasmic mRNAs as well as surveillance of defective nuclear pre-mRNAs, whereas the activity of Rrp6 is expected to be nuclear-specific (reviewed in (Houseley and Tollervey, 2009)). Nuclear and cytoplasmic mRNAs cannot be distinguished in sequence, other than by the presence of introns specifically in nuclear pre-mRNAs. We therefore analyzed spliced mRNAs for relative read coverage over introns and exons. Analysis of reads mapped to intron-exon boundaries (IE) in unspliced pre-mRNA to exon-exon boundaries (EE) in spliced mRNA (Figure 6A; IE/EE) showed substantially higher binding to pre-mRNAs for Rrp6 than for intact Rrp44 or Rrp44-endo, whereas Rrp44-exo showed strongly enhanced pre-mRNA binding. Total binding over introns relative to all mRNAs was also notably higher for Rrp6 than for intact Rrp44 (Figure 6A; Introns/Total mRNA) or the other core exosome components (data not shown), again with strongly enhanced binding by Rrp44-exo. The differences for total introns was less marked than for the IE boundary, probably because Rrp44 and Rrp6 also degrade excised introns following debranching. Comparison of total binding at the 3′ and 5′ splice sites (Figure 6A; 3′SS/5′SS) showed preferential binding to the 5′SS for both Rrp6 and Rrp44, which was particularly marked for Rrp44-exo.

Comparison of individual spliced genes (Figure 6B), confirmed the preferential association of Rrp44 with 5′ regions including the 5′SS. The Rrp44-exo mutant was particularly strongly enriched there and reads frequently extended into the intron, giving rise to the high IE/EE ratio (Figure 6A). In contrast, Rrp6 showed the highest numbers of reads across introns, possibly reflecting a major role in degradation of both excised introns and pre-mRNAs. The coverage of Rrp44-exo over pre-mRNAs and mRNAs was lower (in hits per million) than for wild-type Rrp44, due to the enrichment of Pol III transcripts in the Rrp44-exo data set (Figures 1C and 6C). This may reflect a relative lack of strong secondary structure in the (pre-)mRNAs relative to highly structured Pol III RNAs that are potentially less readily degraded.

A notable feature of the alignments in Figure 6B was the bimodal distribution of intron lengths, coupled with notably higher sequence coverage on the genes with long introns compared to shorter introns in each of the data sets. This was particularly seen over exon 2 sequences, showing that it arises from targeting of the exosome to the pre-mRNAs or, conceivably, the spliced mRNAs. The long intron gene set was dominated by ribosomal proteins, which are more highly transcribed than most genes in the short intron set. These differences in expression levels are probably largely responsible for increased sequence coverage. However, regulated splicing of yeast ribosomal protein genes has been reported (Pleiss et al., 2007), which may be related to their targeting by the surveillance system.

Clustering of mRNAs by pattern of interactions with Rrp44, Rrp44-exo and Rrp6 revealed a class of transcripts preferentially bound by Rrp6. This subclass is enriched for intron-containing genes (p < 1e-4, chi-square test), as well as ribosomal protein genes (p < 1e-10) and ribosome synthesis factors (p < 1e-6) identified by the DAVID functional annotation tool (Dennis et al., 2003) (Figure 6C).

Together these results are consistent with core-independent functions and nuclear localization of Rrp6 and highlight a substantial role for the nuclear exosome in pre-mRNA surveillance and degradation.

**Figure 7:**
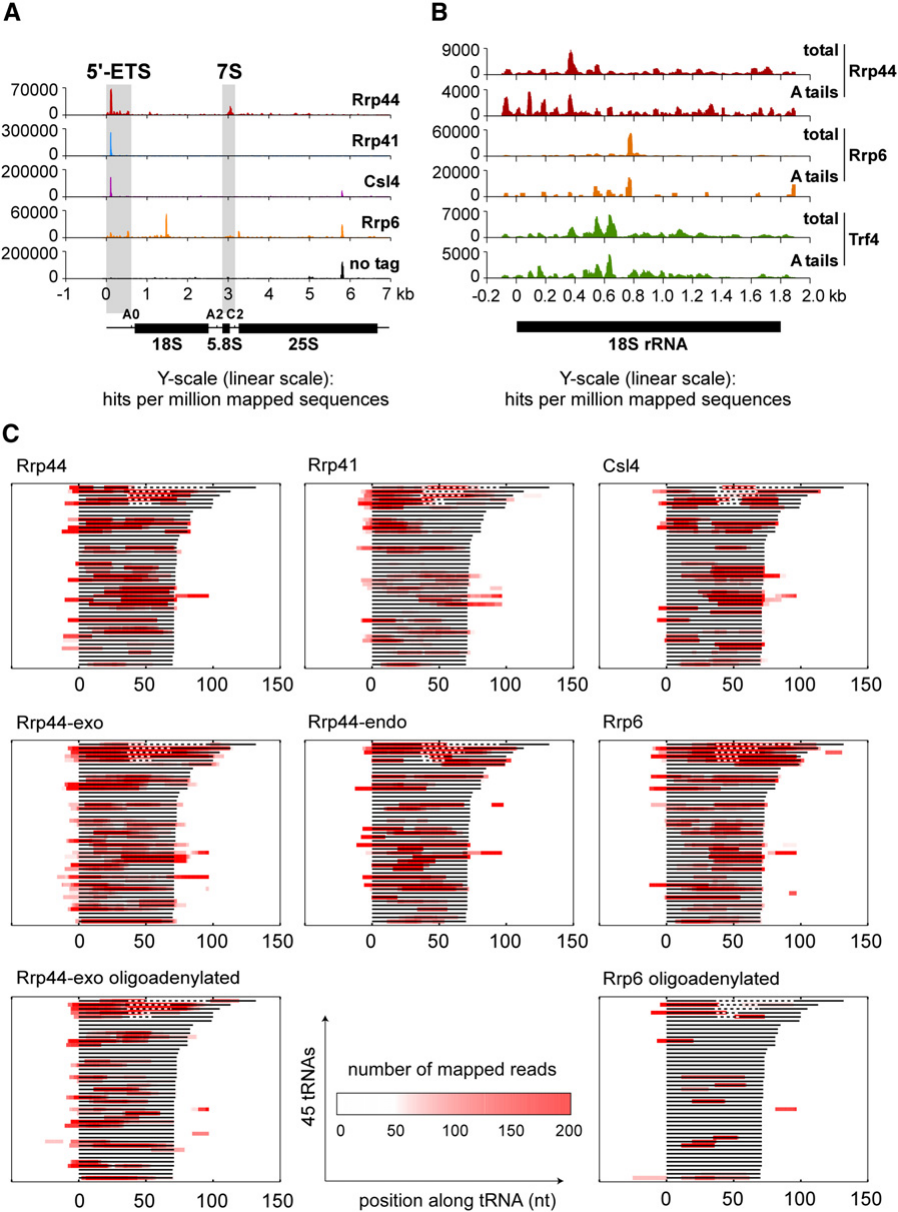
Distribution of High-Throughput Sequencing Reads from Core Exosome, Rrp6, and Trf4 Data Sets over the Pre-rRNA and (Pre-)tRNAs (A) Coverage of high-throughput sequencing reads along the 35S pre-rRNA (6.9kb). The peak around 5.8kb in the 25S rRNA is a background contaminant seen in many experiments (Granneman et al., 2009; Granneman et al., 2010; van Nues et al., 2011). (B) Coverage of reads, either unfiltered (total) or filtered for reads containing 2 or more non-templated As (A tails), from Rrp44, Rrp6 and Trf4 data sets were mapped to the 18S rRNA region of the pre-rRNA. (C) The lines indicate 45 different yeast tRNAs (one for each anticodon family). Dashed lines indicate the presence of introns in the pre-tRNAs. The tRNAs are ranked by length (including intron if present) and aligned at the 5′ end of the mature sequence. Read coverage is indicated by color intensity.

Binding profiles over the entire 35S pre-ribosomal RNA also showed distinctions between Rrp6 and the core exosome (Figure 7A). Over the 5′-ETS, the core exosome components showed similar binding, whereas Rrp6 was clearly distinct. The Rrp44 and Rrp6 hits partially overlap in the ITS2 region, where these proteins function at different steps in the 3′ processing of 7S pre-rRNA (a 3′ extended form of 5.8S rRNA; highlighted in gray in Figure 7A) (Allmang et al., 1999). In contrast, no crosslinking was seen here for Rrp41 or Csl4.

Within the 18S rRNA region (Figure 7B), Rrp6 showed prominent peaks that were absent from the Rrp41 and Csl4 data sets, whereas some of the sites were recovered at lower levels with Rrp44. Previous analyses showed that RNA Pol I is prone to transcription pausing in this region leading to cotranscriptional cleavage of the nascent transcript, which is degraded by Rrp6 and the TRAMP polyadenylation complex (El Hage et al., 2010). Consistent with this, crosslinking to the Trf4 component of the TRAMP complex showed an overlapping peak in 18S (Figure 7B).

To assess the function of the exosome in the surveillance of RNA Pol III transcripts, hit distributions of Rrp44, Rrp6, Rrp41 and Csl4 were compared for each tRNA species (Figure 7C). Sequence reads extended beyond both ends of the mature tRNA (solid lines in Figure 7C) and included intronic sequences (dashed lines in Figure 7C) showing that pre-tRNAs are targets. This can also be seen for combined sets of all spliced and non-spliced tRNAs (Figure S5). Comparison of hit densities confirms the preferential association of pre-tRNAs with Rrp6 relative to the core exosome components (Figure S5). However, increased coverage on 5′ and 3′ extended regions seen for the Rrp44-exo mutant relative to Rrp44 (Figure 7C) demonstrates that many pre-tRNAs are targets for the exonuclease activity of Rrp44. Pre-tRNA recovery was comparable between Rrp44, Rrp41 and Csl4, indicating that these RNAs make direct contacts with the exosome core, consistent with threading through the lumen of the exosome. Oligoadenylated reads recovered with Rrp44-exo were distributed toward the 5′ end relative to total hits. These RNAs must represent truncation products that have been tailed with oligo(A) during degradation. The exosome is heavily dependent on cofactors, including the TRAMP complex. These can only bind the target RNA 5′ to the degrading exosome or Rrp6, since the 3′ end of the RNA is in the active site of the nuclease. We speculate that the processivity of degradation may be impaired when there is insufficient RNA available 5′ to the exosome for cofactors to remain bound. This may lead to stalling, substrate release and oligoadenylation by TRAMP, as outlined in Figure 4D.

## Discussion

The exosome targets a huge variety of RNA substrates, but in most cases it remains unclear how RNAs are specifically targeted to the distinct enzymatic activities associated with the complex. To better understand exosome targeting in budding yeast, we performed highly sensitive in vivo RNA-protein crosslinking studies (CRAC), coupled with deep sequencing, on the exosome-associated nucleases Rrp44 and Rrp6, two structural subunits Rrp41 and Csl4, and the exosome cofactor Trf4.

Increased relative crosslinking to pre-tRNA was seen for the Rrp44-exo mutant relative to wild-type Rrp44. This strongly indicates that the highly structured pre-tRNAs are substrates for the exonuclease activity of Rrp44, which become “stuck” in the mutant. In contrast, pre-mRNAs and mRNAs, which are expected to be relatively unstructured compared to Pol III transcripts, were under-represented in Rrp44-exo data sets. Northern analysis revealed the accumulation of pre-tRNAs in Rrp44-exo strains, whereas levels of mature tRNAs were not clearly affected. This is consistent with reduced surveillance, rather than impaired processing of pre-tRNAs (Wlotzka et al., 2011). Pre-tRNAs did not clearly accumulate in *rrp6Δ* single mutant strains (data not shown and (Copela et al., 2008)), but combinatorial loss of Rrp6 and Trf4 strongly amplified the accumulation of pre-tRNAs relative to the absence of Trf4 alone (Copela et al., 2008). We predict that Rrp6 plays a major role in pre-tRNA surveillance in vivo, but this is redundant with the exonuclease activity of Rrp44 and the core exosome.

Other Pol III transcripts, 5S rRNA, U6 snRNA and scR1 were also preferentially crosslinked to Rrp44-exo, as well as to Rrp6 and Trf4 (Wlotzka et al., 2011). This suggests that Rrp44 and Rrp6 directly cooperate to degrade these RNAs, aided by the TRAMP complex. Supporting this idea, the 3′ truncated form of the 5S rRNA (5S^∗^) seen in Rrp44-exo strains also accumulated in strains lacking Trf4 or Rrp6 (Kadaba et al., 2006) and when interactions between Rrp6 and the core exosome were impaired (Callahan and Butler, 2010). Rrp44-exo strains accumulated mature and 3′ extended (up to 3nt) spliceosomal U6 snRNA, together with 3′ truncated forms of the 5S rRNA (5S^∗^) and scR1 (scR1^∗^) (Copela et al., 2008; Kadaba et al., 2006). Oligoadenylated fragments derived from these RNAs were strongly enriched among Rrp44-exo targets, consistent with Trf4 crosslinking (this study and Wlotzka et al., 2011), strongly indicating that these are surveillance rather than processing targets.

Together, the data suggest that wild-type cells produce excess Pol III transcripts, which are normally turned over by Rrp44 and other nuclear 3′ exonucleases including Rrp6 (Callahan and Butler, 2008; Copela et al., 2008; Kadaba et al., 2006; Schneider et al., 2007). Recent transcriptome-wide tiling microarrays and pulse-chase labeling of pre-tRNAs indicate that more than 50% of tRNA gene transcription fails to generate mature, functional tRNAs (Gudipati et al., 2012). A major pathway of exosome-mediated pre-tRNA turnover that competes with tRNA maturation would be consistent with our crosslinking results. Persistent binding of pre-tRNAs to the exosome in the absence of Rrp44 exonuclease activity very likely contributes to the impaired growth and RNA processing in Rrp44-exo strains. The recent finding that ∼10% of patients suffering from multiple myeloma carry Rrp44-exo mutations (Chapman et al., 2011) suggests that either increased synthesis of RNA Pol III products, or the resulting impaired RNA surveillance can induce malignant transformation in human cells.

Nuclear pre-mRNAs and cytoplasmic mRNAs are both targets for the core exosome, whereas the activity of Rrp6 is predicted to be specific for the nuclear RNAs (reviewed in (Houseley and Tollervey, 2009)). However, these species cannot readily be distinguished in short sequence reads, other than by the presence of the intron. The clearest distinction is therefore the comparison between intron-exon boundaries (IE), which must be part of the unspliced pre-mRNA, and exon-exon boundaries (EE), which can only be present in the spliced mRNA. Among Rrp6 targets, IE hits were around 2 fold more numerous than EE hits, strongly supporting a role for Rrp6 in pre-mRNA surveillance. Consistent with this, analysis of the distribution of Rrp6 reads across spliced genes shows clear enrichment over introns. Cluster analyses of mRNAs showing preferential enrichment in the Rrp6 data sets identified spliced pre-mRNAs but, surprisingly, also found many ribosome synthesis factors. These mRNAs may undergo a significant level of nuclear degradation, possibly as a regulatory mechanism.

Fully functional Rrp44 showed a lower level of sequences over IE boundaries and lower total read coverage over introns, however, both were very substantially increased in the Rrp44-exo mutant. This indicates that Rrp44 is normally actively engaged in degradation of unspliced or partially spliced pre-mRNAs, but these are rapidly and efficiently cleared with little time for crosslinking. Rrp44 showed a high level of crosslinking at the 5′ ends of pre-mRNAs and preferential binding to 5′ splice sites relative to 3′ splice sites. Degradation by the exosome is dependent on cofactors, which must bind 5′ to the complex. Increased crosslinking in the 5′ region may therefore reflect loss of these cofactors leading to slowed degradation.

The Rrp44 sequence coverage over the exons of genes that contain long introns (mainly ribosomal protein genes) was strikingly higher than over genes with shorter introns. This is in agreement with the observation that pre-mRNAs with long introns are preferentially stabilized by loss of Rrp44 function (Gudipati et al., 2012), clearly showing that these are more subject to degradation by the exosome. Whether this is related to the regulated splicing reported for ribosomal protein pre-mRNAs (Pleiss et al., 2007) remains to be determined.

Other Pol II transcripts that are largely degraded in the nucleus include CUTs and SUTs. These transcript classes each showed similar sequence coverage for core exosome and Rrp6. SUTs were designated as “stable un-annotated transcripts” based on a lack of apparent stabilization in the absence of Rrp6 (Xu et al., 2009). However, the similar crosslinking patterns of CUTs and SUTs, and recent microarray analyses in exosome mutant strains (Gudipati et al., 2012), indicate that their degradation pathways are more closely related than their names suggest.

Close functional interactions between Rrp44 and Rrp6 presumably help explain why strains lacking Rrp44 exonuclease activity survive. Although the CTD domain of the Rrp44-exo protein may be tightly and non-productively associated with substrates, the endonucleolytic activity in the N-terminal PIN domain (NTD) of Rrp44 presumably remains competent to cleave these RNAs, providing free 3′ ends for Rrp6 and other exonucleases. Consistent with this model, the split-CRAC data revealed that exonuclease and endonuclease activities of Rrp44 usually act together to degrade RNA substrates. Mapping of the relative binding sites of the Rrp44 NTD and CTD regions combined with analyses of oligoadenylated substrates, leads us to propose a model (Figure 4D) for the role of the PIN domain in releasing stalled exosome substrates. In Rrp44-exo strains, RNAs will be degraded inefficiently, but will still be released from the core exosome by PIN domain cleavage and presented to Rrp6 or other nucleases. In the Rrp44-endo-exo double mutant these substrates may be permanently bound to Rrp44, leading to the accumulation of gridlocked exosome complexes and non-functional RNAs in the cell.

In contrast to pre-tRNAs, other highly structured RNAs that were strongly crosslinked to Rrp6 often showed very few hits in Rrp41 and Csl4 data sets, suggesting that they interact only with Rrp44 and Rrp6, with little or no contact to the remaining core exosome. This was unexpected because in vitro data indicated that many substrates are channeled to Rrp44 through the catalytically inert exosome barrel (Bonneau et al., 2009). Instead, the in vivo crosslinking data on structured RNAs suggest the use of an alternative entry site to the Rrp44 catalytic center, without contacts to the exosome barrel. Such an alternative entry site can be fitted onto the Rrp44-Rrp41-Rrp45 crystal structure (Bonneau et al., 2009). We therefore hypothesize that at least some in vivo substrates are not threaded through the exosome channel. Instead, they could be docked to Rrp44 from the outside of the complex, aided by tethering to Rrp6 and other exosome cofactors. The basis for this distinction remains unclear, but a long (∼33 nt) single-stranded region is required to access the exonuclease domain of Rrp44 via the exosome lumen, whereas much shorter single-stranded regions would be sufficient for direct access to the catalytic sites of Rrp44 or Rrp6.

Despite the apparent cooperation of Rrp44 and Rrp6 on many nuclear surveillance substrates, the comparison of crosslinking sites on individual core exosome subunits with Rrp6 also revealed substrates only enriched in Rrp6 data sets, revealing core-independent Rrp6 functions. One such example is the prominent Rrp6 peak in the 5′-half of the mature 18S rRNA (Figure 7B), which also coincides with a peak of crosslinking by Trf4 (Wlotzka et al., 2011). This corresponds to an RNA polymerase I pause site, at which R-loop formation leads to RNase H cleavage of the nascent transcript (El Hage et al., 2010). We conclude that Rrp6 and the TRAMP complex degrade the cotranscriptionally truncated Pol I primary transcript independently of the core exosome. Rrp6 was reported to localize to the rDNA, interacting with the Nrd1/Nab3 heterodimer and the transcription elongation factors Spt4 and Spt5 (Leporé and Lafontaine, 2011). We therefore speculate that Rrp6 is specifically recruited to the elongating Pol I to survey nascent rRNA transcripts.

## Experimental Procedures

### Strains and Expression Constructs

Growth and handling of *S. cerevisiae* were by standard techniques. Strains were grown at 25°C or 30°C in synthetic dropout (SD) medium containing 0.67% nitrogen base (Difco) and either 2% glucose or 2% galactose.

Yeast strains for crosslinking studies on Trf4, Rrp41, Csl4 and Rrp6 were constructed by standard methods (Gietz et al., 1992) and expressed genomically encoded, C-terminal HTP-tagged (see below) proteins under the control of their endogenous promoter (see Tables S1 and S2). Strains expressing wild-type and mutant forms of Rrp44 were generated by plasmid shuffling of Rrp44 expression constructs into a host strain derived from BY4741, where the genomic RRP44 ORF was precisely deleted (Schneider et al., 2007; Schneider et al., 2009). Rrp44 expression plasmids comprise the RRP44 ORF under control of its endogenous promoter and different C-terminal and/or internal tags (see below). Plasmids designed for split-CRAC contain a PreScission protease cleavage site (PP) inserted between aa 241 and 242 in the RRP44 ORF to allow in vitro cleavage of purified protein, and a His_6_ tag to select the respective cleaved fragment. Point mutations were created using the QuikChange kit (Stratagene). C-terminal tandem affinity purification tags used for basic CRAC and in vivo analyses: HTP: His_6_ - TEV cleavage site (TEV) - two copies of the z-domain of protein A (protA); szz: Streptavidin-binding peptide (Strep-tag II) – TEV – protA. Cleavable expression constructs used for split-CRAC to purify N- and C-terminal fragments: Rrp44 N-terminus: His_6_ - PP inserted at aa 241 + C-terminal TEV – protA; Rrp44 C terminus: PP inserted at aa 241 + C-terminal His6 – TEV – protA.

### Crosslinking and Analysis of Illumina Sequence Data

The CRAC method was performed as previously described (Granneman et al., 2009; Granneman et al., 2011), see Figure 1B for illustration. If not stated otherwise, the same experimental procedure and bioinformatic analyses were applied to all CRAC experiments. To generate RNA-protein crosslinks, actively growing yeast cell cultures in SD medium (OD_600_ ∼0.5) were UV-irradiated in a 1.2 m metal tube (“Megatron”) for 100 s at 254 nm (Granneman et al., 2011). During split-CRAC on Rrp44, purified full-length proteins were first released from the IgG sepharose resin by TEV protease cleavage and then treated for 2 hr at 18°C with PreScission protease (PP). Cleaved N- and C-terminal fragments were then purified on Ni-agarose under standard CRAC denaturing conditions (Granneman et al., 2009). Illumina sequencing data was aligned to the yeast genome using Novoalign (http://www.novocraft.com). Bioinformatics analyses were performed as described (Wlotzka et al., 2011). The primary sequence data are available from the NCBI Gene Expression Omnibus (Edgar et al., 2002) through GEO Series accession number GSE40046. Mapped reads are presented in Table S3.

### RNA Analyses

Yeast RNA extraction and Northern hybridization were performed as described (Tollervey, 1987). Northern signals were visualized by autoradiography or generated by a Fuji FLA-5100 PhosphorImager.

## Supplemental Information

Document S1. Five Figures and Two Tables

Table S3. Locations of Mapped Hits

## References

[bib1] Allmang C., Kufel J., Chanfreau G., Mitchell P., Petfalski E., Tollervey D. (1999). Functions of the exosome in rRNA, snoRNA and snRNA synthesis. EMBO J..

[bib2] Bonneau F., Basquin J., Ebert J., Lorentzen E., Conti E. (2009). The yeast exosome functions as a macromolecular cage to channel RNA substrates for degradation. Cell.

[bib3] Briggs M.W., Burkard K.T., Butler J.S. (1998). Rrp6p, the yeast homologue of the human PM-Scl 100-kDa autoantigen, is essential for efficient 5.8 S rRNA 3′ end formation. J. Biol. Chem..

[bib4] Callahan K.P., Butler J.S. (2008). Evidence for core exosome independent function of the nuclear exoribonuclease Rrp6p. Nucleic Acids Res..

[bib5] Callahan K.P., Butler J.S. (2010). TRAMP complex enhances RNA degradation by the nuclear exosome component Rrp6. J. Biol. Chem..

[bib6] Chapman M.A., Lawrence M.S., Keats J.J., Cibulskis K., Sougnez C., Schinzel A.C., Harview C.L., Brunet J.P., Ahmann G.J., Adli M. (2011). Initial genome sequencing and analysis of multiple myeloma. Nature.

[bib7] Copela L.A., Fernandez C.F., Sherrer R.L., Wolin S.L. (2008). Competition between the Rex1 exonuclease and the La protein affects both Trf4p-mediated RNA quality control and pre-tRNA maturation. RNA.

[bib8] Davis C.A., Ares M.J. (2006). Accumulation of unstable promoter-associated transcripts upon loss of the nuclear exosome subunit Rrp6p in Saccharomyces cerevisiae. Proc. Natl. Acad. Sci. USA.

[bib9] Dennis G., Sherman B.T., Hosack D.A., Yang J., Gao W., Lane H.C., Lempicki R.A. (2003). DAVID: Database for Annotation, Visualization, and Integrated Discovery. Genome Biol..

[bib10] Edgar R., Domrachev M., Lash A.E. (2002). Gene Expression Omnibus: NCBI gene expression and hybridization array data repository. Nucleic Acids Res..

[bib11] El Hage A., French S.L., Beyer A.L., Tollervey D. (2010). Loss of Topoisomerase I leads to R-loop-mediated transcriptional blocks during ribosomal RNA synthesis. Genes Dev..

[bib12] Gietz D., St Jean A., Woods R.A., Schiestl R.H. (1992). Improved method for high efficiency transformation of intact yeast cells. Nucleic Acids Res..

[bib13] Granneman S., Kudla G., Petfalski E., Tollervey D. (2009). Identification of protein binding sites on U3 snoRNA and pre-rRNA by UV cross-linking and high-throughput analysis of cDNAs. Proc. Natl. Acad. Sci. USA.

[bib14] Granneman S., Petfalski E., Swiatkowska A., Tollervey D. (2010). Cracking pre-40S ribosomal subunit structure by systematic analyses of RNA-protein cross-linking. EMBO J..

[bib15] Granneman S., Petfalski E., Tollervey D. (2011). A cluster of ribosome synthesis factors regulate pre-rRNA folding and 5.8S rRNA maturation by the Rat1 exonuclease. EMBO J..

[bib16] Grzechnik P., Kufel J. (2008). Polyadenylation linked to transcription termination directs the processing of snoRNA precursors in yeast. Mol. Cell.

[bib17] Gudipati R.K., Xu Z., Lebreton A., Séraphin B., Steinmetz L.M., Jacquier A., Libri D. (2012). Massive degradation of RNA precursors by the exosome in wild type cells. Mol. Cell.

[bib18] Hogan D.J., Riordan D.P., Gerber A.P., Herschlag D., Brown P.O. (2008). Diverse RNA-binding proteins interact with functionally related sets of RNAs, suggesting an extensive regulatory system. PLoS Biol..

[bib19] Houseley J., Tollervey D. (2009). The many pathways of RNA degradation. Cell.

[bib20] Jamonnak N., Creamer T.J., Darby M.M., Schaughency P., Wheelan S.J., Corden J.L. (2011). Yeast Nrd1, Nab3, and Sen1 transcriptome-wide binding maps suggest multiple roles in post-transcriptional RNA processing. RNA.

[bib21] Kadaba S., Krueger A., Trice T., Krecic A.M., Hinnebusch A.G., Anderson J. (2004). Nuclear surveillance and degradation of hypomodified initiator tRNAMet in S. cerevisiae. Genes Dev..

[bib22] Kadaba S., Wang X., Anderson J.T. (2006). Nuclear RNA surveillance in Saccharomyces cerevisiae: Trf4p-dependent polyadenylation of nascent hypomethylated tRNA and an aberrant form of 5S rRNA. RNA.

[bib23] Kiss D.L., Andrulis E.D. (2010). Genome-wide analysis reveals distinct substrate specificities of Rrp6, Dis3, and core exosome subunits. RNA.

[bib24] Lebreton A., Tomecki R., Dziembowski A., Séraphin B. (2008). Endonucleolytic RNA cleavage by a eukaryotic exosome. Nature.

[bib25] Leporé N., Lafontaine D.L. (2011). A functional interface at the rDNA connects rRNA synthesis, pre-rRNA processing and nucleolar surveillance in budding yeast. PLoS ONE.

[bib26] Liu Q., Greimann J.C., Lima C.D. (2006). Reconstitution, activities, and structure of the eukaryotic RNA exosome. Cell.

[bib27] Lorentzen E., Basquin J., Tomecki R., Dziembowski A., Conti E. (2008). Structure of the active subunit of the yeast exosome core, Rrp44: diverse modes of substrate recruitment in the RNase II nuclease family. Mol. Cell.

[bib28] Malet H., Topf M., Clare D.K., Ebert J., Bonneau F., Basquin J., Drazkowska K., Tomecki R., Dziembowski A., Conti E. (2010). RNA channelling by the eukaryotic exosome. EMBO Rep..

[bib29] Neil H., Malabat C., d’Aubenton-Carafa Y., Xu Z., Steinmetz L.M., Jacquier A. (2009). Widespread bidirectional promoters are the major source of cryptic transcripts in yeast. Nature.

[bib30] Pleiss J.A., Whitworth G.B., Bergkessel M., Guthrie C. (2007). Rapid, transcript-specific changes in splicing in response to environmental stress. Mol. Cell.

[bib31] San Paolo S., Vanacova S., Schenk L., Scherrer T., Blank D., Keller W., Gerber A.P. (2009). Distinct roles of non-canonical poly(A) polymerases in RNA metabolism. PLoS Genet..

[bib32] Schaeffer D., Tsanova B., Barbas A., Reis F.P., Dastidar E.G., Sanchez-Rotunno M., Arraiano C.M., van Hoof A. (2009). The exosome contains domains with specific endoribonuclease, exoribonuclease and cytoplasmic mRNA decay activities. Nat. Struct. Mol. Biol..

[bib33] Schneider C., Anderson J.T., Tollervey D. (2007). The exosome subunit Rrp44 plays a direct role in RNA substrate recognition. Mol. Cell.

[bib34] Schneider C., Leung E., Brown J., Tollervey D. (2009). The N-terminal PIN domain of the exosome subunit Rrp44 harbors endonuclease activity and tethers Rrp44 to the yeast core exosome. Nucleic Acids Res..

[bib35] Tollervey D. (1987). A yeast small nuclear RNA is required for normal processing of pre-ribosomal RNA. EMBO J..

[bib36] van Nues R.W., Granneman S., Kudla G., Sloan K.E., Chicken M., Tollervey D., Watkins N.J. (2011). Box C/D snoRNP catalysed methylation is aided by additional pre-rRNA base-pairing. EMBO J..

[bib37] Vanácová S., Wolf J., Martin G., Blank D., Dettwiler S., Friedlein A., Langen H., Keith G., Keller W. (2005). A new yeast poly(A) polymerase complex involved in RNA quality control. PLoS Biol..

[bib38] Wlotzka W., Kudla G., Granneman S., Tollervey D. (2011). The nuclear RNA polymerase II surveillance system targets polymerase III transcripts. EMBO J..

[bib39] Wyers F., Rougemaille M., Badis G., Rousselle J.-C., Dufour M.-E., Boulay J., Régnault B., Devaux F., Namane A., Séraphin B. (2005). Cryptic pol II transcripts are degraded by a nuclear quality control pathway involving a new poly(A) polymerase. Cell.

[bib40] Xu Z., Wei W., Gagneur J., Perocchi F., Clauder-Münster S., Camblong J., Guffanti E., Stutz F., Huber W., Steinmetz L.M. (2009). Bidirectional promoters generate pervasive transcription in yeast. Nature.

[bib41] Zhang C., Darnell R.B. (2011). Mapping in vivo protein-RNA interactions at single-nucleotide resolution from HITS-CLIP data. Nat. Biotechnol..

[bib42] Zuo Y., Vincent H.A., Zhang J., Wang Y., Deutscher M.P., Malhotra A. (2006). Structural basis for processivity and single-strand specificity of RNase II. Mol. Cell.

