# Supplementary material for: Transcriptome-wide Analysis of Exosome Targets
Source: Mol Cell. 2012 Nov 9;48(3-3):422–33. doi: 10.1016/j.molcel.2012.08.013 (PMC3526797; doi:10.1016/j.molcel.2012.08.013)
Supplement: Document S1. Five Figures and Two Tables [file mmc1.pdf]

Molecular Cell, Volume *48*

Supplemental Information

Transcriptome-wide Analysis of Exosome Targets

Claudia Schneider, Grzegorz Kudla, Wiebke Wlotzka, Alex Tuck, and David Tollervey

Table S1: Oligonucleotides used in these studies.
Oligonucleotides are indicated (5'-3'), rN indicates RNA nucleotide

Construction of Rrp44-HTP plasmids, derived from Rrp44-szz (Schneider et al., 2009)

Rrp44-genF	ACGTACTCGAGGAATATATCCTTTTGAACTGGAGTG
Rrp44-gen(URA)R	ACGTACTCGAGGCTGGATGGGAAGCGTACC
Rrp44-His$_6$-overlap-F	GAGAAGCCACCATCACCATCACCATACTGCTAGCGAGAATTTG
Rrp44-His$_6$-overlap-R	GTTAAAATCCATGGAAAAGAGAAGCCACCATCACCATCACCAT

Insertion of a PreScission Protease (PP) cleavage site into Rrp44-HTP or Rrp44-szz plasmids

Rrp44-PP-F	CTGGAAGTTCTGTTCCAGGGGCCCGATAAAGATCTTGAAAGGG ACACATTTTCAG
Rrp44-PP-R	GGGCCCCTGGAACAGAACTTCCAGAAAAGAATCCATTTGAGGAATA GAGTCTCTG
Rrp44-His$_6$-PP-F	CACCATCACCATCACCATCTGGAAGTTCTGTTCCAGGGGCCCGA TAAAGATCTTGAAAGGGACACATTTTCAG
Rrp44-His$_6$-PP-R	GGGCCCCTGGAACAGAACTTCCAGATGGTGATGGTGATGGTG AAAAGAATCCATTTGAGGAATAGAGTCTCTG

Construction of HTP-tagged yeast strains

Rrp41-HTP-F	GGAAACATGCTCAGAAAAGAGTCAGTAACGCCTCTGCTAGGG AGCACCATCACCATCACC
Rrp41-TAP-R	ACTTTTATATAAACAGTGGCAATTAATGGCGTTTTTTATTTACG ACTCACTATAGGG
Csl4-HTP-F	TTACAGGCGCTACAGAAAAGCGCAAATGTGCCAAACCTTTTG AGCACCATCACCATCACC
Csl4-TAP-R	TATATACGCGTCTATATGCACTGTAGATAAGCTGTTACATATA CGACTCACTATAGGG
Rrp6-HTP-F	AAAGAGGAGGCCTGCCGCCAAAGGTAAGAATCTGTCATTTAA AAGGTCCATGGAGCACCATC
Rrp6-TAP-R	TAACTCCATGACACAGATATTCGATTAGATGAATTTAGAGGTC TTAATACGACTCACTATAGGG
pBS1539-HTP-URA	plasmid for HTP tagging, Granneman *et al.*, 2009

CRAC 5' linkers (IDT)

5'L	5InvddT/GTTCArGrArGrUrUrCrUrArCrArGrUrCrCrGrArCrGrArUrC
L5c	5InvddT/ACACrGrArCrGrCrUrCrUrUrCrCrGrArUrCrUrGrA
L5d	5InvddT/ACACrGrArCrGrCrUrCrUrUrCrCrGrArUrCrUrArCrArArGrC
L5e	5InvddT/ACACrGrArCrGrCrUrCrUrUrCrCrGrArUrCrUrCrArCrArGrC
L5f	5InvddT/ACACrGrArCrGrCrUrCrUrUrCrCrGrArUrCrUrGrCrGrArGrC
L5Aa	5InvddT/ACACrGrArCrGrCrUrCrUrUrCrCrGrArUrCrUrNrNrNrUrArArGrC
L5Ad	5InvddT/ACACrGrArCrGrCrUrCrUrUrCrCrGrArUrCrUrNrNrNrCrGrCrUrUrArGrC

CRAC 3′ pre-activated, adenylated cloning linker (IDT)

miRCat-33^{TM} 3'-L	rAppTGGAATTCTCGGGTGCCAAGG/ddC/

CRAC RT and PCR

miRCat-33™ RT	CCTTGGCACCCGAGAATT
PCR-F1	AATGATACTGCGACCACCGACAGGTTCAGAGTTCTACAGTCCGA
PCR-R1	CAAGCAGAAGACGGCATACGA
PCR-F2	AATGATACGGCGACCACCGAGATCTACACTCTTTCCCTACACGA CGCTCTTCCGATCT
PCR-R2	CAAGCAGAAGACGGCATACGAGATCGGTCTCGGCATTCCTGGC CTTGGCACCCGAGAATTCC

Northern probes

#304 anti-tRNA^{Pro}(UGG) mature	ACCCAGGGCCTCTCG
#305 anti-tRNA^{Trp}(CCA) mature	AACCTGCAACCCTTCGA
W331 anti-tRNA^{Arg}(UCU) mature	GGGGTCGAACCCATAATCTT
#250 anti-scR1	ATCCCGGCCGCCTCCATCAC
#261 anti-U6 snRNA	AAAACGAAATAAATTCTTTGTAAAAC

Table S2: Yeast strains used in these studies.

BY4741	*MATa; his3Δ1; leu2Δ0; lys2Δ0; ura3Δ0*	Euroscarf
Δrrp44 shuffle strain	*MATa; his3Δ1; leu2Δ0; lys2Δ0; ura3Δ0; rrp44Δ::kanMX6; [pRS316/RRP44-szz]*	(Schneider et al., 2009)
Gal::rrp44	*MATa; his3Δ1; leu2Δ0; lys2Δ0; ura3Δ0, His3MX6-pGAL1-3HA::rrp44*	(Houseley and Tollervey, 2006)
Trf4-HTP	*MATa; his3Δ1; leu2Δ0; lys2Δ0; ura3Δ0; TRF4-HTP-URA3*	(Wlotzka et al., 2011)
Rrp6-HTP	*MATa; his3Δ1; leu2Δ0; lys2Δ0; ura3Δ0; RRP6-HTP-URA3*	This study
Csl4-HTP	*MATa; his3Δ1; leu2Δ0; lys2Δ0; ura3Δ0; CSL4-HTP-URA3*	This study
Rrp41-HTP	*MATa; his3Δ1; leu2Δ0; lys2Δ0; ura3Δ0; RRP41-HTP-URA3*	This study

Supplementary References

Gietz, D., St. Jean, A., Woods, R.A., and Schiestl, R.H. (1992). Improved method for high efficiency transformation of intact yeast cells. Nucleic Acids Res. *20*, 1425.
Granneman, S., Kudla, G., Petfalski, E., and Tollervey, D. (2009). Identification of protein binding sites on U3 snoRNA and pre-rRNA by UV cross-linking and high throughput analysis of cDNAs. Proc. Natl. Acad. Sci. U.S.A. *106*, 9613-9818.
Granneman, S., Petfalski, E., and Tollervey, D. (2011). A cluster of ribosome synthesis factors regulate 5.8S rRNA maturation. EMBO J. *30*, 4006-4019.
Houseley, J., and Tollervey, D. (2006). Yeast Trf5p is a nuclear poly(A) polymerase. EMBO Rep. *7*, 205-211.
Schneider, C., Anderson, J.T., and Tollervey, D. (2007). The exosome subunit Rrp44 plays a direct role in RNA substrate recognition. Mol. Cell *27*, 324-331.
Schneider, C., Leung, E., Brown, J., and Tollervey, D. (2009). The N-terminal PIN domain of the exosome subunit Rrp44 harbors endonuclease activity and tethers Rrp44 to the yeast core exosome. Nucleic Acids Res *37*, 1127-1140.
Wlotzka, W., Kudla, G., Granneman, S., and Tollervey, D. (2011). The nuclear RNA polymerase II surveillance system targets polymerase III transcripts. EMBO J. *30*, 1790-1803.

Table S3 (Excel file):

Hit densities on functionally grouped RNAs recovered with all individual biological replicate experiments

Dataset used for making the heatmaps presented in Figures 1, 5, 6 and S1. Numbers of hits per million mapped reads are shown.

Supplementary Figures:

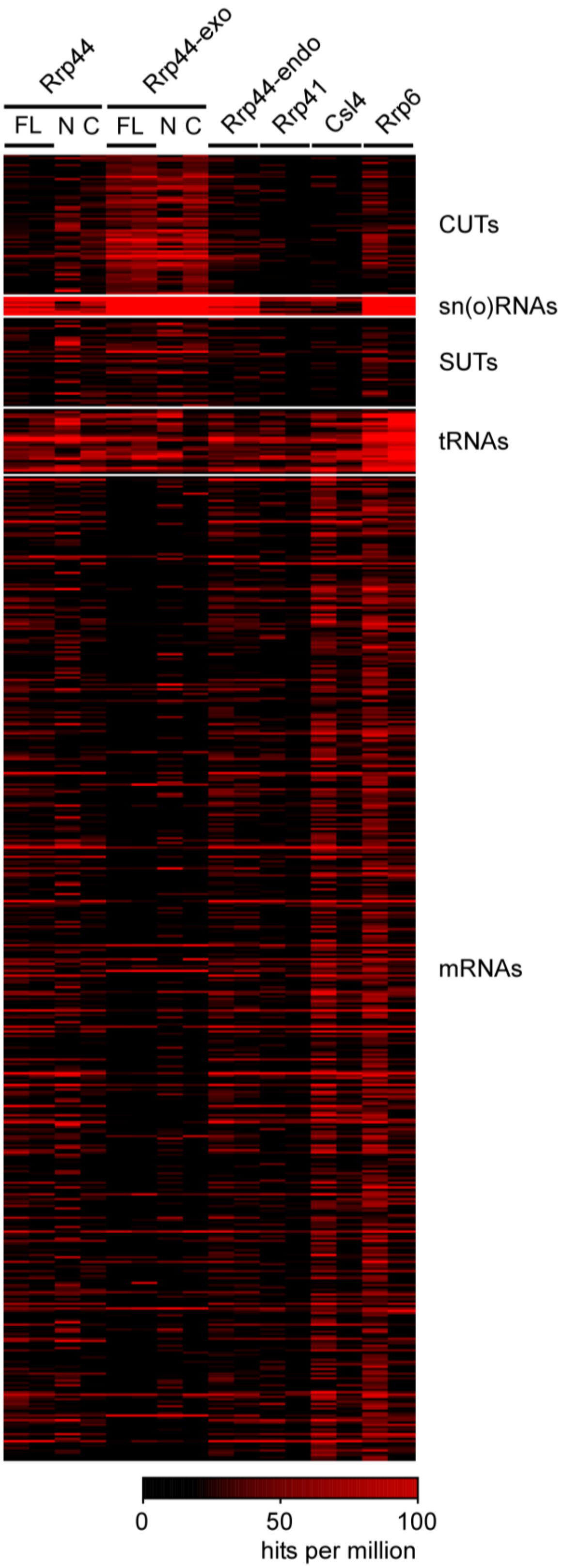

Figure S1: Heat maps for main substrate groups and all individual biological replicate experiments. Hits per million mapped reads of recovered RNAs are shown in shades of red.

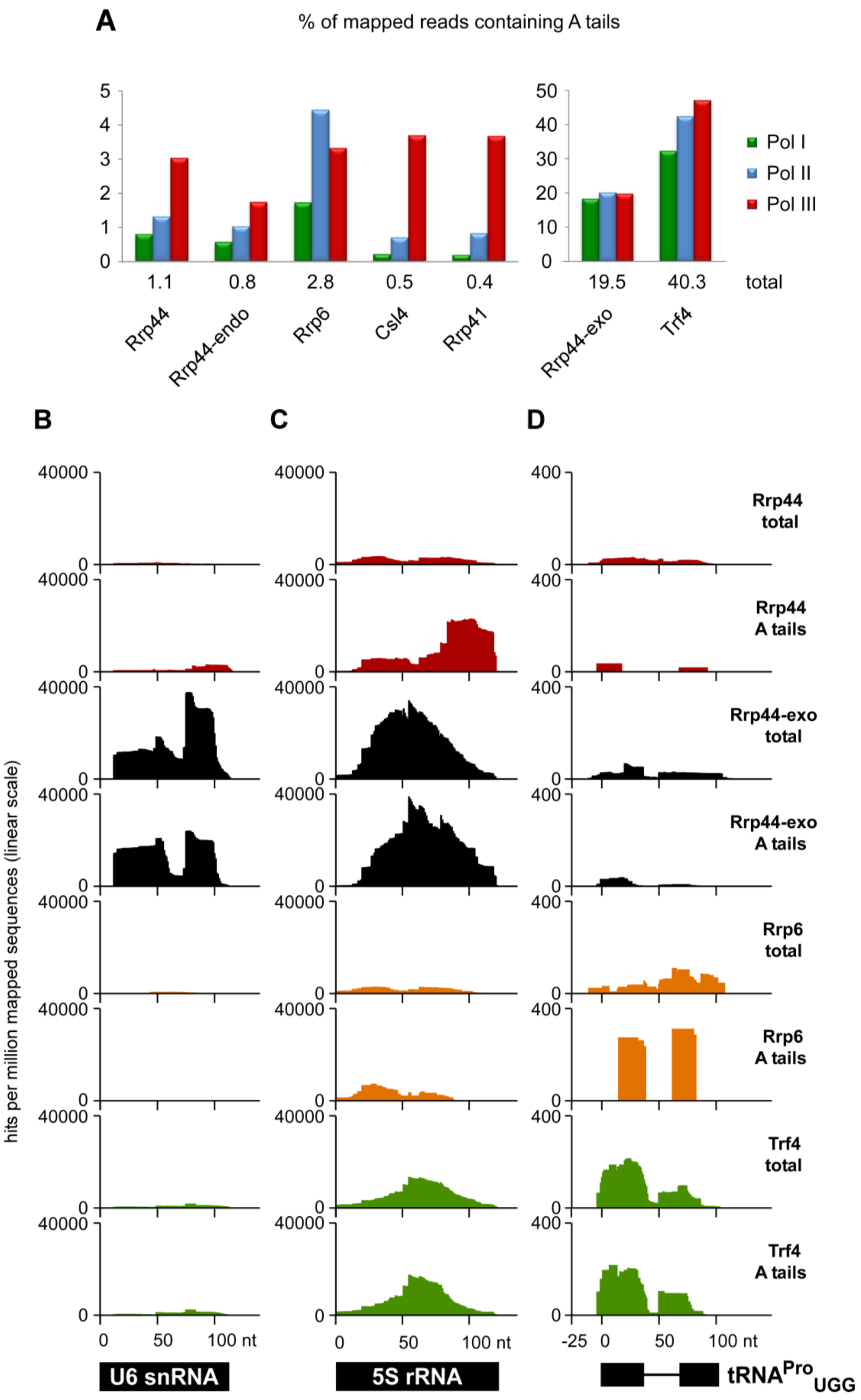

A
% of mapped reads containing A tails
5
4
3
2
1
0
50
40
30
20
10
0
Pol I
Pol II
Pol III
1.1
0.8
2.8
0.5
0.4
19.5
40.3
total
Rrp44
Rrp44-endo
Rrp6
Csl4
Rrp41
Rrp44-exo
Trf4
B
C
D
hits per million mapped sequences (linear scale)
40000
0
400
Rrp44 total
Rrp44 A tails
Rrp44-exo total
Rrp44-exo A tails
Rrp6 total
Rrp6 A tails
Trf4 total
Trf4 A tails
0
50
100 nt
-25
U6 snRNA
5S rRNA
tRNAPro UGG

Figure S2

Figure S2:

A: Frequencies of non-templated terminal oligo(A) sequence reads in core exosome, Rrp6 and Trf4 datasets. Datasets are either filtered for total reads, or for Pol I, Pol II and Pol III transcripts, that contain 2 or more non-templated As.

B, C, D: Oligoadenylated sequence reads derived from representative Pol III RNAs

Densities of high-throughput sequencing reads, either unfiltered (total) or filtered for reads containing 2 or more non-templated As (A-tails), from Rrp44, Rrp44-exo, Rrp6 and Trf4 datasets were mapped to the U6 snRNA (B), 5S rRNA (C) and $tRNA^{Pro}_{UGG}$ (D).

A

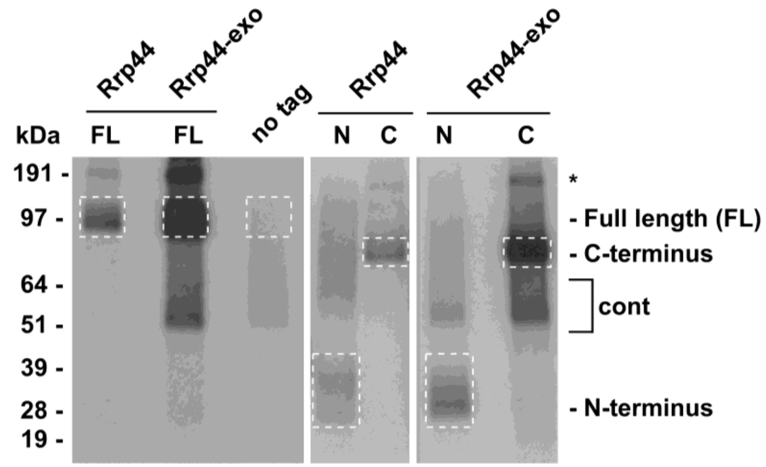

B

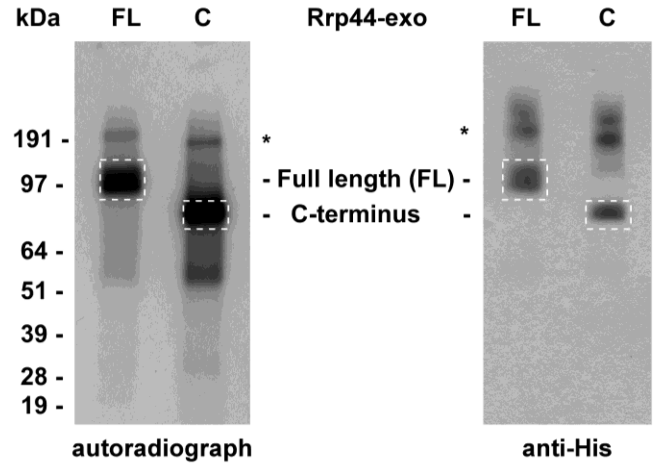

Figure S3: Separation of full length Rrp44 and cleaved fragments

A: Autoradiography of a denaturing SDS PAGE separating full length Rrp44 and N- and C-terminal fragments. Proteins purified from yeast strains expressing either wild type Rrp44 or the Rrp44-exo mutant are visualized by labeling of crosslinked RNA fragments. No tag: untagged strain; cont: common contaminant ~55 kDa band (Granneman *et al.*, 2009).

B: Full length Rrp44-exo and the cleaved C-terminal fragment are visualized by autoradiography (left, see panel A) or immunoblotting using an anti-His antibody (right).

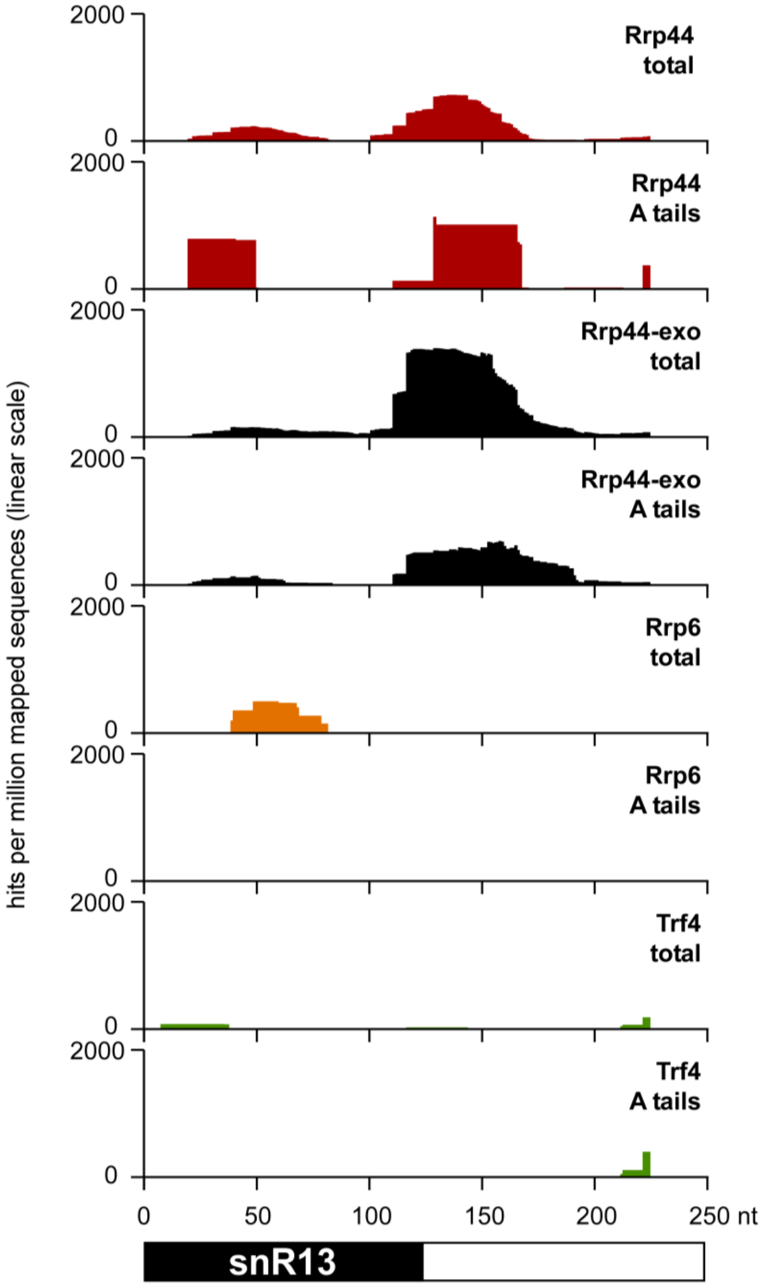

Figure S4: Functional overlap between Rrp44 and Rrp6 on snR13

Densities of high-throughput sequencing reads, either unfiltered (total) or filtered for reads containing 2 or more non-templated As (A-tails), from Rrp44, Rrp44-exo, Rrp6 and Trf4 datasets were mapped to the box C/D snoRNA snR13 (1-124 nt, black box) and downstream regions. The snoRNA terminator elements I and II are located at nt 125-190 (I) and nt 190-249 (II).

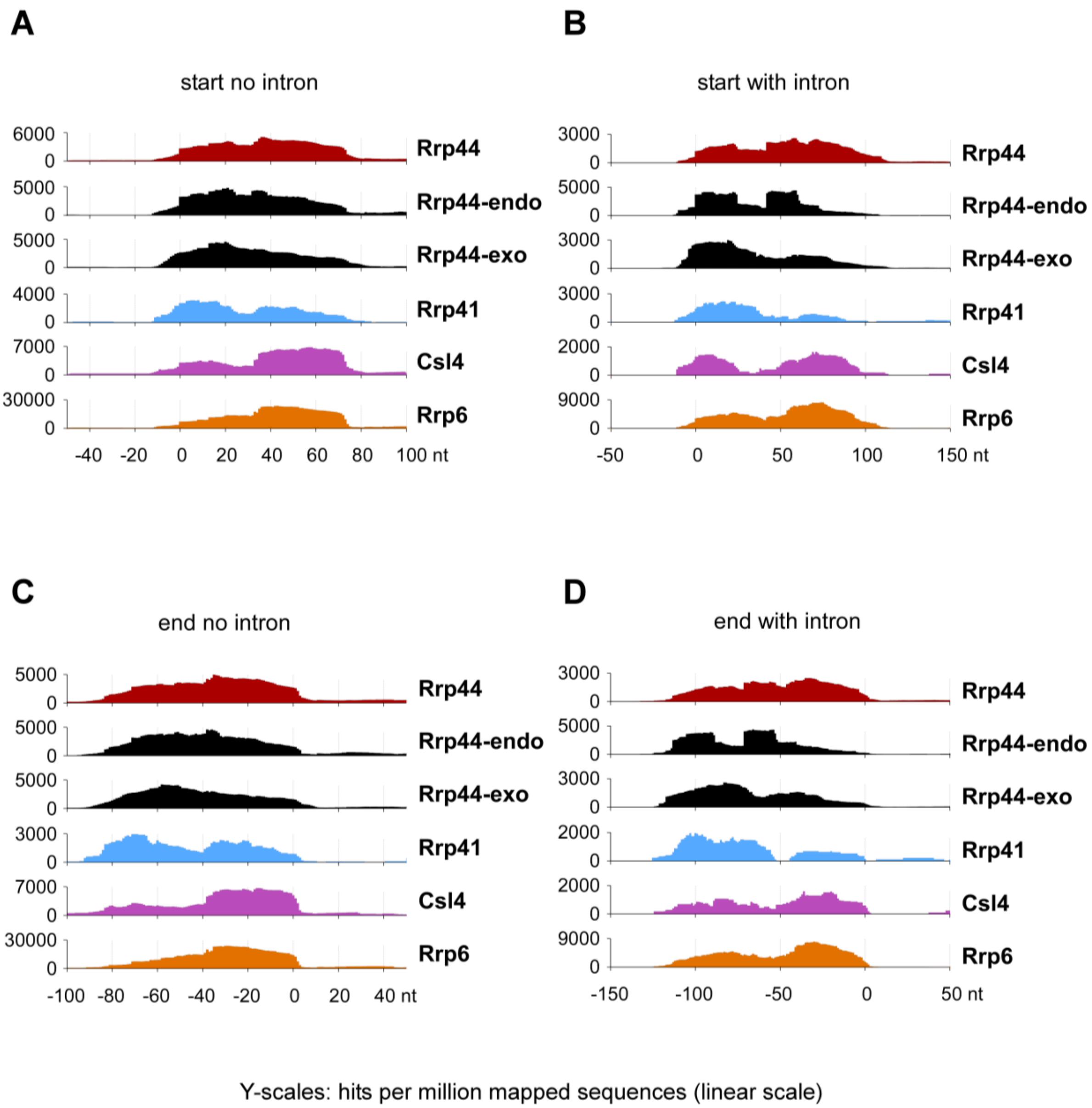

Figure S5: Distribution of high-throughput sequencing reads from core exosome and Rrp6 datasets over all yeast tRNA genes.

All yeast tRNA genes were filtered by the presence or absence of an intron and aligned at either 5' or 3' end of the mature tRNAs (indicated as position 0 on the plots). Total hit densities per million mapped reads are indicated for the proteins tested. Note that different scales are used in the Y-axis, reflecting differences in overall tRNA association for the different proteins.
